# Efficacy and safety in a 4-year follow-up of the ELEVATE-TN study comparing acalabrutinib with or without obinutuzumab versus obinutuzumab plus chlorambucil in treatment-naïve chronic lymphocytic leukemia

**DOI:** 10.1038/s41375-021-01485-x

**Published:** 2022-01-01

**Authors:** Jeff P. Sharman, Miklos Egyed, Wojciech Jurczak, Alan Skarbnik, John M. Pagel, Ian W. Flinn, Manali Kamdar, Talha Munir, Renata Walewska, Gillian Corbett, Laura Maria Fogliatto, Yair Herishanu, Versha Banerji, Steven Coutre, George Follows, Patricia Walker, Karin Karlsson, Paolo Ghia, Ann Janssens, Florence Cymbalista, Jennifer A. Woyach, Emmanuelle Ferrant, William G. Wierda, Veerendra Munugalavadla, Ting Yu, Min Hui Wang, John C. Byrd

**Affiliations:** 1 Willamette Valley Cancer Institute and Research Center, Eugene, OR USA; 2 Somogy County Mór Kaposi General Hospital, Kaposvár, Hungary; 3 Maria Skłodowska-Curie National Research Institute of Oncology, Krakow, Poland; 4 Novant Health Cancer Institute, Charlotte, NC USA; 5 Swedish Cancer Institute, Center for Blood Disorders and Stem Cell Transplantation, Seattle, WA USA; 6 Sarah Cannon Research Institute and Tennessee Oncology, Nashville, TN USA; 7 University of Colorado Cancer Center, Aurora, CO USA; 8 Haematology, Haematological Malignancy Diagnostic Service (HMDS), St. James’s Institute of Oncology, Leeds, UK; 9 Cancer Care, University Hospitals Dorset, Bournemouth, UK; 10 Tauranga Hospital, Tauranga, New Zealand; 11 Hospital de Clinicas de Porto Alegre, Porto Alegre, Brazil; 12 Tel Aviv Sourasky Medical Center, Tel Aviv, Israel; 13 Departments of Internal Medicine, Biochemistry & Medical Genetics, Max Rady College of Medicine, Rady Faculty of Health Sciences, University of Manitoba and CancerCare Manitoba Research Institute, Winnipeg, MB Canada; 14 Stanford University School of Medicine, Stanford, CA USA; 15 Department of Haematology, Addenbrooke’s Hospital NHS Trust, Cambridge, UK; 16 Peninsula Health and Peninsula Private Hospital, Frankston, Melbourne, VIC Australia; 17 Skåne University Hospital, Lund, Sweden; 18 Università Vita-Salute San Raffaele and IRCCS Ospedale San Raffaele, Milano, Italy; 19 University Hospitals Leuven, Leuven, Belgium; 20 Bobigny: Hématologie, CHU Avicennes, Bobigny, France; 21 The Ohio State University Comprehensive Cancer Center, Columbus, OH USA; 22 Hospices Civils de Lyon, Centre Hospitalier Lyon Sud, Service d’Hématologie Clinique, Pierre-Bénite, France; 23 Department of Leukemia, Division of Cancer Medicine, MD Anderson Cancer Center, Houston, TX USA; 24 AstraZeneca, South San Francisco, CA USA

**Keywords:** Targeted therapies, Chronic lymphocytic leukaemia, Combination drug therapy

## To the Editor

Bruton tyrosine kinase (BTK) inhibitors have improved chronic lymphocytic leukemia (CLL) outcomes and offer a chemotherapy-free option [1]. The BTK inhibitor ibrutinib, alone or with a CD20 antibody, demonstrated better efficacy versus chemoimmunotherapy in treatment-naïve (TN) CLL [2–4]. However, cardiovascular toxicity is a concern with continuous ibrutinib use [5, 6].

Acalabrutinib is a next-generation, selective BTK inhibitor approved for CLL/small lymphocytic leukemia (SLL). Acalabrutinib, alone or with obinutuzumab, showed favorable efficacy in clinical trials [7, 8]. ELEVATE-TN demonstrated superior efficacy for acalabrutinib-obinutuzumab versus obinutuzumab-chlorambucil with acceptable tolerability in TN CLL [9]. We report 4-year follow-up results from ELEVATE-TN.

ELEVATE-TN is a phase 3, randomized, multicenter, open-label study (NCT02475681) that enrolled patients aged ≥65 years, or 18–65 years with comorbidities (Cumulative Illness Rating Scale-Geriatric score >6, creatinine clearance 30–69 mL/min by Cockcroft-Gault), who had TN CLL or SLL requiring treatment, Eastern Cooperative Oncology Group performance status score of ≤2, and adequate hematologic, hepatic, and renal function [9]. Patients were randomized (1:1:1) to acalabrutinib 100 mg twice daily (until disease progression or unacceptable toxicity) with or without obinutuzumab (fixed-duration, up to 6 cycles) or obinutuzumab plus chlorambucil (up to 6 cycles). Crossover to acalabrutinib monotherapy was permitted in patients who progressed on obinutuzumab-chlorambucil. The primary study endpoint was independent review committee (IRC)-assessed progression-free survival (PFS). After primary analysis, PFS was investigator-assessed. Key secondary/exploratory endpoints were investigator-assessed PFS, investigator-assessed overall response rate (ORR), overall survival (OS), undetectable minimal residual disease (uMRD) rate, and safety. The study was not powered to compare acalabrutinib versus acalabrutinib-obinutuzumab. Informed consent was obtained from all patients before enrollment. Study details were previously published [9].

In total, 535 patients were randomized (acalabrutinib-obinutuzumab, *n* = 179; acalabrutinib, *n* = 179; obinutuzumab-chlorambucil, *n* = 177). Median age was 70 years (range, 41.0–91.0); 14% had del(17)(p13.1) and/or mutated *TP53* and 63% had unmutated immunoglobulin heavy chain variable (IGHV) gene (Supplementary Table 1).

At a median follow-up of 46.9 months (range, 0.0–59.4), treatment was ongoing in 74.9% (*n* = 134) and 69.3% (*n* = 124) of patients in the acalabrutinib-obinutuzumab and acalabrutinib monotherapy arms, respectively (Supplementary Table 2). Sixty-nine patients (39.0%) in the obinutuzumab-chlorambucil arm had crossed over to acalabrutinib. Overall, 25.1% of acalabrutinib-obinutuzumab patients and 30.7% of acalabrutinib patients discontinued treatment; 22.6% of obinutuzumab-chlorambucil patients did not complete therapy. The most common reason for treatment discontinuation (acalabrutinib-obinutuzumab, acalabrutinib, and obinutuzumab-chlorambucil) was adverse events (AEs; 12.8%, 12.3%, and 14.7%, respectively).

**Fig. 1:**
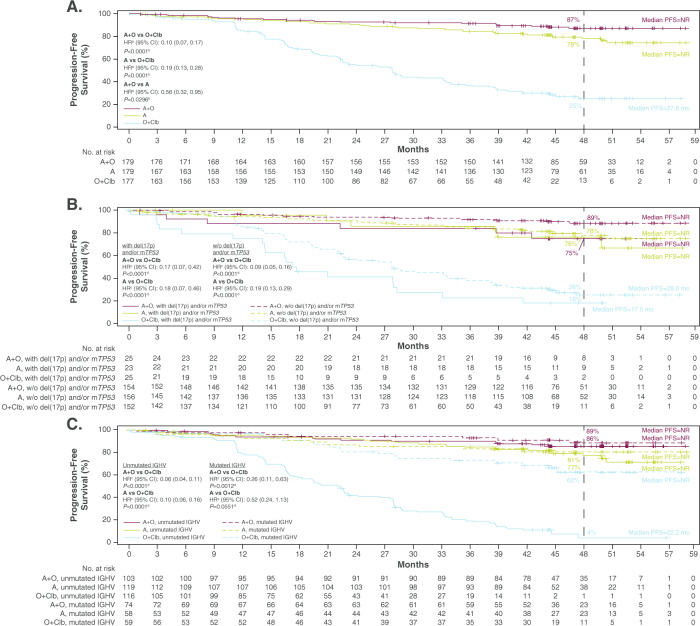
Investigator-assessed progression-free survival (A) overall, (B) by del(17)(p13.1) and/or mutated *TP53* status, and (C) by IGHV mutation status. ^a^Hazard ratio was based on stratified Cox-Proportional-Hazards model; ^b^*P* value was based on stratified log-rank test; ^c^Hazard ratio was based on unstratified Cox-Proportional-Hazards model. ^d^*P* value was based on unstratified log-rank test. A acalabrutinib, CI confidence interval, Clb chlorambucil, HR hazard ratio, IGHV immunoglobulin heavy chain variable region, m*TP53* mutated *TP53*, NR not reached, O obinutuzumab, PFS progression-free survival, w/o without.

Median investigator-assessed PFS was not reached (acalabrutinib-containing arms) versus 27.8 months for obinutuzumab-chlorambucil (both *P* < 0.0001; Fig. 1A). In a post hoc analysis, prolonged PFS also was observed with acalabrutinib-obinutuzumab versus acalabrutinib (*P* = 0.0296; Fig. 1A); however, the study was not sufficiently powered for this comparison. The PFS benefit of acalabrutinib-containing regimens was consistent in high-risk genomic subgroups. In patients with del(17)(p13.1) and/or mutated *TP53*, median PFS was not reached (acalabrutinib-containing arms) versus 17.5 months for obinutuzumab-chlorambucil (both *P* < 0.0001; Fig. 1B); similar results were seen in patients with only del(17)(p13.1) (Supplementary Fig. 1). In patients with unmutated IGHV, median PFS was not reached (acalabrutinib-containing arms) versus 22.2 months for obinutuzumab-chlorambucil (both *P* < 0.0001); median PFS was not reached in any treatment arm in patients with mutated IGHV (Fig. 1C). Estimated 48-month PFS rates overall were 87.0% for acalabrutinib-obinutuzumab, 77.9% for acalabrutinib, and 25.1% for obinutuzumab-chlorambucil. In the acalabrutinib-obinutuzumab and acalabrutinib monotherapy arms, 48-month PFS rates were 74.8% and 76.2%, respectively, for patients with del(17)(p13.1) and/or mutated *TP53*, and 85.7% and 77.1% for patients with unmutated IGHV.

Median OS was not reached in any treatment arm. Fewer deaths occurred in patients receiving acalabrutinib-obinutuzumab versus obinutuzumab-chlorambucil, but the difference was not statistically significant (HR: 0.50; 95% CI, 0.25, 1.02; *P* = 0.0604; Supplementary Fig. 2). While the OS HR for acalabrutinib-obinutuzumab versus acalabrutinib in a post hoc analysis was noteworthy (HR: 0.53; 95% CI, 0.26, 1.06), the difference between the two acalabrutinib-containing arms was not statistically significant (*P* = 0.0836). Estimated 48-month OS rates were 92.9% for acalabrutinib-obinutuzumab, 87.6% for acalabrutinib, and 88.0% for obinutuzumab-chlorambucil.

The ORR was significantly higher with acalabrutinib-obinutuzumab (96.1% [*n* = 172/179]; 95% CI, 92.1, 98.1) versus obinutuzumab-chlorambucil (82.5% [*n* = 146/177]; 95% CI, 76.2, 87.4; *P* < 0.0001; Supplementary Fig. 3A). The ORR with acalabrutinib (89.9% [*n* = 161/179]; 95% CI, 84.7, 93.5) also was significantly higher versus obinutuzumab-chlorambucil (*P* = 0.035). The complete response (CR) rate, including CR with incomplete hematologic recovery (CRi), was higher with acalabrutinib-obinutuzumab (30.7% [*n* = 55/179]) versus obinutuzumab-chlorambucil (13.0% [*n* = 23/177]) and versus acalabrutinib (post hoc; 11.2% [n = 20/179]). Comparing the acalabrutinib-obinutuzumab and acalabrutinib monotherapy arms, CR + CRi rates were 32.0% and 13.0%, respectively, for patients with del(17)(p13.1) and/or mutated *TP53*, and 28.2% and 12.6% for patients with unmutated IGHV. Sustained uMRD rates based on the last two MRD assessments are shown in Supplementary Fig. 3B.

Median treatment exposure was 46.6 months for acalabrutinib-obinutuzumab and 45.7 months for acalabrutinib monotherapy (Table 1); no new safety signals were observed. The most common any-grade AEs (≥30%) were diarrhea, headache, and neutropenia for acalabrutinib-obinutuzumab; diarrhea and headache for acalabrutinib monotherapy; and neutropenia, infusion-related reaction, and nausea for obinutuzumab-chlorambucil (Table 1). AEs occurring more frequently in the acalabrutinib-containing arms included headache, diarrhea, fatigue, arthralgia, cough, and upper respiratory tract infection. Headaches, while common, were typically low grade; none led to treatment discontinuation. Among patients receiving acalabrutinib-obinutuzumab, neutropenia, fatigue, and arthralgia were more frequent relative to acalabrutinib alone. The obinutuzumab-chlorambucil arm had more frequent neutropenia, nausea, and infusion-related reactions relative to both acalabrutinib-containing arms, though differences in AE reporting could be due to the longer treatment exposure in the acalabrutinib-containing arms versus the comparator arm. In the acalabrutinib-containing arms, most of the common AEs decreased in incidence over time, and most events occurred more predominantly during the first year of treatment (Supplementary Table 3). Incidence and time to onset of AEs leading to discontinuation of acalabrutinib-containing treatment are described in Supplementary Table 4.

**Table 1:** Common adverse events (AEs) and selected AEs of interest.

	A + O (*n* = 178)		A (*n* = 179)		O + Clb (*n* = 169)	
Treatment exposure, median (range), months	46.6 (2.3–58.6)		45.7 (0.3–59.3)		5.6 (0.9–7.4)	
Common AEs (in ≥ 25% of patients [any grade] in any group), *n* (%)						
	Any grade	Grade ≥3	Any grade	Grade ≥3	Any grade	Grade ≥3
Diarrhea	73 (41.0)	9 (5.1)	72 (40.2)	1 (0.6)	36 (21.3)	3 (1.8)
Headache	71 (39.9)	2 (1.1)	68 (38.0)	2 (1.1)	20 (11.8)	0
Neutropenia	60 (33.7)	55 (30.9)	22 (12.3)	20 (11.2)	76 (45.0)	70 (41.4)
Fatigue	50 (28.1)	4 (2.2)	39 (21.8)	2 (1.1)	30 (17.8)	2 (1.2)
Arthralgia	47 (26.4)	2 (1.1)	35 (19.6)	2 (1.1)	8 (4.7)	2 (1.2)
Cough	46 (25.8)	1 (0.6)	40 (22.3)	1 (0.6)	15 (8.9)	0
URTI	44 (24.7)	4 (2.2)	46 (25.7)	0	16 (9.5)	1 (0.6)
Nausea	41 (23.0)	0	41 (22.9)	0	53 (31.4)	0
IRR	25 (14.0)	5 (2.8)	0	0	68 (40.2)	10 (5.9)
Selected events of clinical interest, *n* (%)						
Cardiac events^a^	37 (20.8)	14 (7.9)^b^	34 (19.0)	15 (8.4)^c^	13 (7.7)	3 (1.8)
Atrial fibrillation/flutter	7 (3.9)	1 (0.6)	11 (6.1)	2 (1.1)	1 (0.6)	0
Bleeding	84 (47.2)	5 (2.8)	75 (41.9)	5 (2.8)	20 (11.8)	0
Major bleeding^d^	7 (3.9)	5 (2.8)	7 (3.9)	5 (2.8)	2 (1.2)	0
Hypertension	14 (7.9)	6 (3.4)	13 (7.3)	5 (2.8)	7 (4.1)	6 (3.6)
Infections	134 (75.3)	42 (23.6)	132 (73.7)	29 (16.2)	75 (44.4)	14 (8.3)
SPMs	28 (15.7)	13 (7.3)	24 (13.4)	5 (2.8)	7 (4.1)	3 (1.8)
Excluding NMS	15 (8.4)	10 (5.6)	11 (6.1)	4 (2.2)	3 (1.8)	2 (1.2)

*A* acalabrutinib, *AE* adverse event, *Clb* chlorambucil, *IRR* infusion-related reaction, *NMS* non-melanoma skin, *O* obinutuzumab, *SPMs* secondary primary malignancies, *URTI* upper respiratory tract infection.

^a^Cardiac events that occurred in >1 patient (any grade; other than atrial fibrillation) in any group include angina pectoris, palpitations, atrioventricular block complete, myocardial ischemia, tachycardia, bradycardia, cardiac failure, left ventricular failure, myocardial infarction, pericardial effusion, acute myocardial infarction, and supraventricular tachycardia.

^b^Cardiac events (grade ≥3) that occurred in >1 patient (other than atrial fibrillation) include atrioventricular block complete (*n* = 3), angina pectoris (*n* = 2), myocardial ischemia (*n* = 2), and myocardial infarction (*n* = 2).

^c^Cardiac events (grade ≥3) that occurred in >1 patient (other than atrial fibrillation) include acute myocardial infarction (*n* = 3), cardiac failure (*n* = 2), and myocardial infarction (*n* = 2).

^d^Defined as any serious or grade ≥3 hemorrhagic event, or any-grade hemorrhagic event in the central nervous system.

Events of clinical interest (ECIs), including cardiovascular events, were similar in both acalabrutinib arms (Table 1). In addition, the cumulative incidences of atrial fibrillation and hypertension over time were low and similar across treatment groups (Supplementary Fig. 4).

With a median follow-up of 46.9 months, the efficacy and safety of acalabrutinib plus obinutuzumab and acalabrutinib monotherapy were maintained with low rates of treatment discontinuation. Median PFS was not reached for either acalabrutinib-containing arm, and PFS continued to be significantly longer for both acalabrutinib-containing arms versus obinutuzumab-chlorambucil. Consistent with the primary report [9], the acalabrutinib-containing arms continued to demonstrate significantly greater PFS benefits versus obinutuzumab-chlorambucil in high-risk genomic subgroups, including del(17)(p13.1) and/or mutated *TP53* and unmutated IGHV, with longer-term treatment. Of note, the estimated PFS rate at 48 months trended in favor of the acalabrutinib combination versus acalabrutinib monotherapy, consistent with findings from preclinical studies demonstrating that, in contrast to ibrutinib, acalabrutinib does not interfere with the anti-tumor immune-mediated mechanisms of anti-CD20 monoclonal antibodies [10, 11]. In the acalabrutinib-containing arms, the CR/CRi rate increased from the primary analysis at 28.3 months (acalabrutinib-obinutuzumab: 24.0%; acalabrutinib: 7.8% [9]) to the current report at a follow-up of 4 years (30.7% and 11.2%, respectively). In high-risk subgroups, CR/CRi rates were numerically higher with the acalabrutinib combination versus monotherapy; however, the study was not powered for this comparison. Further research is needed to assess the efficacy benefits of acalabrutinib-obinutuzumab combination therapy. With longer-term follow-up, the tolerability profile of the acalabrutinib-containing arms was consistent with that of the primary analysis. Incidences of the most common AEs, such as headache, diarrhea, neutropenia, and fatigue, were generally unchanged or saw a slight increase from the primary analysis [9].

Though cross-trial comparisons are limited, the efficacy results from this study are aligned with those from the iLLUMINATE study of ibrutinib-obinutuzumab in a similar patient population at a median follow-up of 31.3 months [4]. In that study, median PFS (assessed by IRC) was not reached; the estimated 30-month PFS rate was 79% with ibrutinib-obinutuzumab. Atrial fibrillation and hypertension rates with ibrutinib-obinutuzumab (12 and 17%, respectively) in iLLUMINATE [4] were higher than the atrial fibrillation/flutter and hypertension rates reported with acalabrutinib-obinutuzumab in the present study (4 and 8%). Discontinuation due to AEs was similar with ibrutinib-obinutuzumab (16%) in the iLLUMINATE study and with acalabrutinib-obinutuzumab in the present study (13%). By comparison, a head-to-head study of acalabrutinib versus ibrutinib (ELEVATE-RR; NCT02477696) at a median follow-up of 40.9 months demonstrated non-inferiority for PFS (primary endpoint) and a statistically significantly lower incidence of atrial fibrillation/flutter with acalabrutinib versus ibrutinib (9% vs 16%, respectively) in patients with previously treated CLL [12]. In ELEVATE-RR, hypertension incidence was also statistically higher with ibrutinib versus acalabrutinib (23% vs 9%).

Based on these updated results, ELEVATE-TN shows continued efficacy at 4 years and a significant PFS benefit in the acalabrutinib-containing arms regardless of high-risk status. PFS benefit is seen particularly with acalabrutinib-obinutuzumab, although this combination resulted in a higher incidence of AEs compared with acalabrutinib monotherapy. No new safety signals were observed with acalabrutinib-containing treatment with longer-term follow-up. The safety of acalabrutinib-containing treatment was consistent with the primary analysis [9], with a low incidence of ECIs, particularly cardiovascular AEs (atrial fibrillation/flutter and hypertension) and low rates of treatment discontinuation despite longer treatment exposure. Findings illustrate the flexibility to tailor acalabrutinib treatment as monotherapy or combination treatment and support acalabrutinib as a combination partner with obinutuzumab in the first-line CLL setting.

## Supplementary information

Supplement

## References

[1] Wen T, Wang J, Shi Y, Qian H, Liu P. Inhibitors targeting Bruton’s tyrosine kinase in cancers: drug development advances. Leukemia. 2021;35:312–32. 33122850 10.1038/s41375-020-01072-6 PMC7862069

[2] Woyach JA, Ruppert AS, Heerema NA, Zhao W, Booth AM, Ding W, et al. Ibrutinib regimens versus chemoimmunotherapy in older patients with untreated CLL. N Engl J Med. 2018;379:2517–28. 30501481 10.1056/NEJMoa1812836 PMC6325637

[3] Shanafelt TD, Wang XV, Kay NE, Hanson CA, O’Brien S, Barrientos J, et al. Ibrutinib-rituximab or chemoimmunotherapy for chronic lymphocytic leukemia. N Engl J Med. 2019;381:432–43. 31365801 10.1056/NEJMoa1817073 PMC6908306

[4] Moreno C, Greil R, Demirkan F, Tedeschi A, Anz B, Larratt L, et al. Ibrutinib plus obinutuzumab versus chlorambucil plus obinutuzumab in first-line treatment of chronic lymphocytic leukaemia (iLLUMINATE): a multicentre, randomised, open-label, phase 3 trial. Lancet Oncol. 2019;20:43–56. 30522969 10.1016/S1470-2045(18)30788-5

[5] Pellegrini L, Novak U, Andres M, Suter T, Nagler M. Risk of bleeding complications and atrial fibrillation associated with ibrutinib treatment: a systematic review and meta-analysis. Crit Rev Oncol Hematol. 2021;159:103238. 33515702 10.1016/j.critrevonc.2021.103238

[6] Archibald WJ, Rabe KG, Kabat BF, Herrmann J, Ding W, Kay NE, et al. Atrial fibrillation in patients with chronic lymphocytic leukemia (CLL) treated with ibrutinib: risk prediction, management, and clinical outcomes. Ann Hematol. 2021;100:143–55. 32488603 10.1007/s00277-020-04094-3 PMC8772341

[7] Byrd JC, Woyach JA, Furman RR, Martin P, O’Brien S, Brown JR, et al. Acalabrutinib in treatment-naïve chronic lymphocytic leukemia. Blood. 2021;137:3327–38. 33786588 10.1182/blood.2020009617 PMC8670015

[8] Woyach JA, Blachly JS, Rogers KA, Bhat SA, Jianfar M, Lozanski G, et al. Acalabrutinib plus obinutuzumab in treatment-naive and relapsed/refractory chronic lymphocytic leukemia. Cancer Disco. 2020;10:394–405. 10.1158/2159-8290.CD-19-1130 PMC8176161 31915195

[9] Sharman JP, Egyed M, Jurczak W, Skarbnik A, Pagel JM, Kamdar M, et al. Acalabrutinib with or without obinutuzumab versus chlorambucil and obinutuzmab for treatment-naive chronic lymphocytic leukaemia (ELEVATE TN): a randomised, controlled, phase 3 trial. Lancet. 2020;395:1278–91. 32305093 10.1016/S0140-6736(20)30262-2 PMC8151619

[10] Golay J, Ubiali G, Introna M. The specific Bruton tyrosine kinase inhibitor acalabrutinib (ACP-196) shows favorable in vitro activity against chronic lymphocytic leukemia B cells with CD20 antibodies. Haematologica. 2017;102:e400–e403. 28642301 10.3324/haematol.2017.169334 PMC5622871

[11] Chu CC, Pinney JJ, Blick-Nitko SK, Baran AM, Peterson DR, Whitehead HE, et al. Ibrutinib off-target inhibition inhibits antibody-dependent cellular phagocytosis but not efferocytosis of CLL cells [abstract]. Blood. 2020;136 Suppl 1:45.

[12] Byrd JC, Hillmen P, Ghia P, Kater AP, Chanan-Khan A, Furman RR, et al. Acalabrutinib versus ibrutinib in previously treated chronic lymphocytic leukemia: results of the first randomized phase III trial. J Clin Oncol. 2021;39:3441–52. 34310172 10.1200/JCO.21.01210 PMC8547923

