# Correction: Efficacy and safety in a 4-year follow-up of the ELEVATE-TN study comparing acalabrutinib with or without obinutuzumab versus obinutuzumab plus chlorambucil in treatment-naïve chronic lymphocytic leukemia

**DOI:** 10.1038/s41375-024-02487-1

**Published:** 2024-12-03

**Authors:** Jeff P. Sharman, Miklos Egyed, Wojciech Jurczak, Alan Skarbnik, John M. Pagel, Ian W. Flinn, Manali Kamdar, Talha Munir, Renata Walewska, Gillian Corbett, Laura Maria Fogliatto, Yair Herishanu, Versha Banerji, Steven Coutre, George Follows, Patricia Walker, Karin Karlsson, Paolo Ghia, Ann Janssens, Florence Cymbalista, Jennifer A. Woyach, Emmanuelle Ferrant, William G. Wierda, Veerendra Munugalavadla, Ting Yu, Min Hui Wang, John C. Byrd

**Affiliations:** 1 Willamette Valley Cancer Institute and Research Center, Eugene, OR USA; 2 Somogy County Mór Kaposi General Hospital, Kaposvár, Hungary; 3 Maria Skłodowska-Curie National Research Institute of Oncology, Krakow, Poland; 4 Novant Health Cancer Institute, Charlotte, NC USA; 5 Swedish Cancer Institute, Center for Blood Disorders and Stem Cell Transplantation, Seattle, WA USA; 6 Sarah Cannon Research Institute and Tennessee Oncology, Nashville, TN USA; 7 University of Colorado Cancer Center, Aurora, CO USA; 8 Haematology, Haematological Malignancy Diagnostic Service (HMDS), St. James’s Institute of Oncology, Leeds, UK; 9 Cancer Care, University Hospitals Dorset, Bournemouth, UK; 10 Tauranga Hospital, Tauranga, New Zealand; 11 Hospital de Clinicas de Porto Alegre, Porto Alegre, Brazil; 12 Tel Aviv Sourasky Medical Center, Tel Aviv, Israel; 13 Departments of Internal Medicine, Biochemistry & Medical Genetics, Max Rady College of Medicine, Rady Faculty of Health Sciences, University of Manitoba and CancerCare Manitoba Research Institute, Winnipeg, MB Canada; 14 Stanford University School of Medicine, Stanford, CA USA; 15 Department of Haematology, Addenbrooke’s Hospital NHS Trust, Cambridge, UK; 16 Peninsula Health and Peninsula Private Hospital, Frankston, Melbourne, VIC Australia; 17 Skåne University Hospital, Lund, Sweden; 18 Università Vita-Salute San Raffaele and IRCCS Ospedale San Raffaele, Milano, Italy; 19 University Hospitals Leuven, Leuven, Belgium; 20 Bobigny: Hématologie, CHU Avicennes, Bobigny, France; 21 The Ohio State University Comprehensive Cancer Center, Columbus, OH USA; 22 Hospices Civils de Lyon, Centre Hospitalier Lyon Sud, Service d’Hématologie Clinique, Pierre-Bénite, France; 23 Department of Leukemia, Division of Cancer Medicine, MD Anderson Cancer Center, Houston, TX USA; 24 AstraZeneca, South San Francisco, CA USA

**Keywords:** Targeted therapies, Chronic lymphocytic leukaemia, Combination drug therapy

Correction to: *Leukemia* 10.1038/s41375-021-01485-x, published online 01 January 2022

The wrong Supplementary file was originally published with this article; it has now been replaced with the correct file. The original article has been corrected.

## Supplementary information

Supplementary Information

