# Supplementary material for: Efficacy and safety in a 4-year follow-up of the ELEVATE-TN study comparing acalabrutinib with or without obinutuzumab versus obinutuzumab plus chlorambucil in treatment-naïve chronic lymphocytic leukemia
Source: Leukemia. 2022 Jan 1;36(4):1171–5. doi: 10.1038/s41375-021-01485-x (PMC8979808; doi:10.1038/s41375-021-01485-x)
Supplement: Supplementary file 1 — Supplement [file 41375_2021_1485_MOESM1_ESM.pdf]

SUPPLEMENTAL INFORMATION TO:

Efficacy and Safety in a 4-Year Follow-Up of the ELEVATE-TN Study Comparing Acalabrutinib With or Without Obinutuzumab Versus Obinutuzumab Plus Chlorambucil in Treatment-Naïve Chronic Lymphocytic Leukemia

Jeff P. Sharman[1], Miklos Egyed[2], Wojciech Jurczak[3], Alan Skarbnik[4], John M. Pagel[5], Ian W. Flinn[6], Manali Kamdar[7], Talha Munir[8], Renata Walewska[9], Gillian Corbett[10], Laura Maria Fogliatto[11], Yair Herishanu[12], Versha Banerji[13], Steven Coutre[14], George Follows[15], Patricia Walker[16], Karin Karlsson[17], Paolo Ghia[18], Ann Janssens[19], Florence Cymbalista[20], Jennifer A. Woyach[21], Emmanuelle Ferrant[22], William G. Wierda[23], Veerendra Munugalavadla[24], Ting Yu[24], Min Hui Wang[24], John C. Byrd[21]

[1]Willamette Valley Cancer Institute and Research Center, Eugene, Oregon, United States; [2]Somogy County Mór Kaposi General Hospital, Kaposvár, Hungary; [3]Maria Skłodowska-Curie National Research Institute of Oncology, Krakow, Poland; [4]Novant Health Cancer Institute, Charlotte, North Carolina, United States; [5]Swedish Cancer Institute, Center for Blood Disorders and Stem Cell Transplantation, Seattle, Washington, United States; [6]Sarah Cannon Research Institute and Tennessee Oncology, Nashville, Tennessee, United States; [7]University of Colorado Cancer Center, Aurora, Colorado, United States; [8]Haematology, Haematological Malignancy Diagnostic Service (HMDS), St. James's Institute of Oncology, Leeds, United Kingdom; [9]Cancer Care, University Hospitals Dorset, Bournemouth, United Kingdom; [10]Tauranga Hospital, Tauranga, New Zealand; [11]Hospital de Clinicas de Porto Alegre, Porto Alegre, Brazil;

[12]Tel Aviv Sourasky Medical Center, Tel Aviv, Israel; [13]Departments of Internal Medicine, Biochemistry & Medical Genetics, Max Rady College of Medicine, Rady Faculty of Health Sciences, University of Manitoba and CancerCare Manitoba Research Institute, Winnipeg, Canada; [14]Stanford University School of Medicine, Stanford, California, United States; [15]Department of Haematology, Addenbrooke's Hospital NHS Trust, Cambridge, United Kingdom; [16]Peninsula Health and Peninsula Private Hospital, Frankston, Melbourne, Australia; [17]Skåne University Hospital, Lund, Sweden; [18]Università Vita-Salute San Raffaele and IRCCS Ospedale San Raffaele, Milano, Italy; [19]University Hospitals Leuven, Leuven, Belgium; [20]Bobigny: Hématologie, CHU Avicennes, Bobigny, France; [21]The Ohio State University Comprehensive Cancer Center, Columbus, Ohio, United States; [22]Hospices Civils de Lyon, Centre Hospitalier Lyon Sud, Service d'Hématologie Clinique, Pierre-Bénite, France; [23]Department of Leukemia, Division of Cancer Medicine, MD Anderson Cancer Center, Houston, Texas, United States; [24]AstraZeneca, South San Francisco, California, United States

SUPPLEMENTAL TABLES

Supplemental Table 1. Demographics and Baseline Characteristics

**Characteristic**	**A+O (n=179)**	**A (n=179)**	**O+Clb (n=177)**
Age, median (range), y	70 (41–88)	70 (44–87)	71 (46–91)
Male sex	111 (62.0)	111 (62.0)	106 (59.9)
ECOG PS score			
0–1	169 (94.4)	165 (92.2)	167 (94.4)
2	10 (5.6)	14 (7.8)	10 (5.6)
Bulky disease ≥5 cm	46 (25.7)	68 (38.0)	54 (30.5)
Rai stage			
III	47 (26.3)[a]	51 (28.5)[a]	40 (22.6)
IV	38 (21.2)	37 (20.7)	38 (21.5)
Cytogenetic subgroup			
Del(17)(p13.1)	17 (9.5)	16 (8.9)	16 (9.0)
Del(17)(p13.1) and/or mutated *TP53*	25 (14.0)	23 (12.8)	25 (14.1)
Del(11q)	31 (17.3)	31 (17.3)	33 (18.6)
Complex karyotype[b]	29 (16.2)	31 (17.3)	32 (18.1)
Mutated *TP53*	21 (11.7)	19 (10.6)	21 (11.9)
Unmutated IGHV	103 (57.5)	119 (66.5)	116 (65.5)

Data are n (%) unless otherwise specified.
[a]The proportion of patients with Rai stage III in the A+O and A arms differed from that reported in the primary publication (48 [26.8%] and 50 [27.9%], respectively[1]) due to lack of database lock at interim analysis and the potential for site-level changes post-interim analysis.
[b]Patients with ≥3 cytogenetic abnormalities.
A, acalabrutinib; Clb, chlorambucil; ECOG PS, Eastern Cooperative Oncology Group performance status; IGHV, immunoglobulin heavy chain variable region; O, obinutuzumab.

1. Sharman JP, Egyed M, Jurczak W, Skarbnik A, Pagel JM, Kamdar M, et al. Acalabrutinib with or without obinutuzumab versus chlorambucil and obinutuzmab for treatment-naive chronic lymphocytic leukaemia (ELEVATE TN): a randomised, controlled, phase 3 trial. Lancet 2020; **395**: 1278-1291.

Supplemental Table 2. Patient Disposition and Exposure

**Characteristic**	**A+O (n=179)**	**A (n=179)**	**O+Clb (n=177)**
Treated with ≥1 dose of study drug	179 (100.0)	178 (99.4)	169 (95.5)
Randomized but not treated	0	1 (0.6)	8 (4.5)
Treatment status			
Ongoing	134 (74.9)	124 (69.3)	0
Completed regimen	NA	NA	137 (77.4)
Discontinued regimen	45 (25.1)	55 (30.7)	40 (22.6)
Death	2 (1.1)[a]	7 (3.9)[b]	3 (1.7)[c]
Adverse event	23 (12.8)	22 (12.3)	26 (14.7)
Lost to follow-up	0	1 (0.6)	1 (0.6)
CLL progressive disease	8 (4.5)	14 (7.8)	3 (1.7)
Withdrawal of consent	2 (1.1)	2 (1.1)	6 (3.4)
Investigator's discretion	5 (2.8)	6 (3.4)	0
Other	5 (2.8)[d]	3 (1.7)[e]	1 (0.6)[f]
Treatment exposure, median (range), months	46.6 (2.3–58.6)	45.7 (0.3–59.3)	5.6 (0.9–7.4)

Data are n (%) unless otherwise specified.
[a]Due to adverse event (n=2); [b]Due to adverse event (n=6) and cerebrovascular accident (n=1); [c]Due to adverse event (n=2) and car accident (n=1); [d]Includes patient decision (n=2), treatment interruption due to disease improvement (n=1), treatment interruption >28 days (n=1), and bleeding risk concerns with clopidogrel bisulfate and acetylsalicylic acid concomitant treatment (n=1); [e]Includes patient decision (n=1), CML diagnosis (n=1), and drug initially held due to macular edema and new lung cancer growth found at time of restart (n=1); [f]Did not meet eligibility criteria (post-randomization determination by sponsor; n=1).
A, acalabrutinib; Clb, chlorambucil; CLL, chronic lymphocytic leukemia; NA, not applicable; O, obinutuzumab.

Supplemental Table 3. AEs Over Time (Any Grade) in ≥25% of Patients in Any Treatment Arm

AE, n (%)	Treatment	≤1 Year[a]	>1–2 Years[b]	>2–3 Years[c]	>3–4 Years[d]	>4 Years[e]
Diarrhea	A+O	59 (33.1)	27 (16.9)	30 (20.0)	27 (18.8)	12 (15.0)
	A	56 (31.3)	26 (16.9)	18 (12.3)	19 (14.2)	10 (13.2)
	O+Clb[f]	36 (21.3)	0	0	0	0
Headache	A+O	65 (36.5)	17 (10.6)	17 (11.3)	14 (9.7)	4 (5.0)
	A	65 (36.3)	24 (15.6)	23 (15.8)	22 (16.4)	10 (13.2)
	O+Clb[f]	20 (11.8)	0	0	0	0
Neutropenia	A+O	51 (28.7)	18 (11.3)	6 (4.0)	5 (3.5)	1 (1.3)
	A	14 (7.8)	8 (5.2)	2 (1.4)	5 (3.7)	1 (1.3)
	O+Clb[f]	76 (45.0)	0	0	0	0
Fatigue	A+O	47 (26.4)	24 (15.0)	20 (13.3)	17 (11.8)	8 (10.0)
	A	27 (15.1)	24 (15.6)	23 (15.8)	19 (14.2)	11 (14.5)
	O+Clb[f]	30 (17.8)	0	0	0	0
Arthralgia	A+O	29 (16.3)	27 (16.9)	26 (17.3)	28 (19.4)	18 (22.5)
	A	22 (12.3)	19 (12.3)	13 (8.9)	16 (11.9)	5 (6.6)
	O+Clb[f]	8 (4.7)	0	0	0	0
Cough	A+O	27 (15.2)	19 (11.9)	18 (12.0)	12 (8.3)	4 (5.0)
	A	23 (12.8)	15 (9.7)	16 (11.0)	13 (9.7)	6 (7.9)
	O+Clb[f]	15 (8.9)	0	0	0	0
URTI	A+O	18 (10.1)	19 (11.9)	21 (14.0)	5 (3.5)	2 (2.5)
	A	24 (13.4)	14 (9.1)	15 (10.3)	10 (7.5)	2 (2.6)
	O+Clb[f]	16 (9.5)	0	0	0	0
Nausea	A+O	34 (19.1)	16 (10.0)	15 (10.0)	12 (8.3)	5 (6.3)
	A	31 (17.3)	15 (9.7)	9 (6.2)	5 (3.7)	3 (3.9)
	O+Clb[f]	53 (31.4)	0	0	0	0
IRR	A+O	25 (14.0)	0	0	0	0
	A	0	0	0	0	0
	O+Clb[f]	68 (40.2)	0	0	0	0

A patient with multiple severity grades for a given AE was counted only once under the maximum severity. Multiple onsets of the same AE within a specific yearly interval were counted once, and the same AE term continuing across several yearly intervals was counted in each of the intervals.
[a]A+O (n=178), A (n=179), O+Clb (n=169); [b]A+O (n=160), A (n=154), O+Clb (n=0); [c]A+O (n=150), A (n=146), O+Clb (n=0); [d]A+O (n=144), A (n=134), O+Clb (n=0); [e]A+O (n=80), A (n=76), O+Clb (n=0); [f]O+Clb treatment was for fixed duration of 6 cycles.
A, acalabrutinib; AE, adverse event; Clb, chlorambucil; IRR, infusion-related reaction; O, obinutuzumab; URTI, upper respiratory tract infection.

Supplemental Table 4. Incidence and Time to Onset of AEs (Any Grade) Leading to Acalabrutinib Discontinuation

	**A+O (n=178)**	**A (n=179)**
Time to onset of AE leading to discontinuation, months		
Mean (SD)	16.9 (14.7)	16.3 (18.3)
Median (range)	9.6 (1.7–44.8)	8.6 (0.2–57.4)
Time to onset of AE leading to discontinuation		
<3 months	3 (1.7)	8 (4.5)
3–6 months	5 (2.8)	1 (0.6)
6–9 months	4 (2.2)	4 (2.2)
9–12 months	1 (0.6)	2 (1.1)
12–24 months	3 (1.7)	2 (1.1)
24–36 months	4 (2.2)	3 (1.7)
36–48 months	4 (2.2)	2 (1.1)
>48 months	0	2 (1.1)
AE leading to treatment discontinuation		
Abdominal distension	1 (0.6)	0
Acute kidney injury	1 (0.6)	0
Acute myeloid leukemia	0	1 (0.6)
Acute myocardial infarction	0	1 (0.6)

Bladder neoplasm	1 (0.6)	0
Brain injury	0	1 (0.6)
Brain neoplasm	0	1 (0.6)
Bronchopulmonary aspergillosis	0	1 (0.6)
Cardiac failure	0	1 (0.6)
Cardiac tamponade	0	1 (0.6)
Chronic myeloid leukemia	0	1 (0.6)
Chronic obstructive pulmonary disease	1 (0.6)	0
Coronary artery stenosis	0	1 (0.6)
Delirium	0	1 (0.6)
Disseminated cryptococcosis	0	1 (0.6)
Fatigue	1 (0.6)	1 (0.6)
Febrile neutropenia	1 (0.6)	0
Gastrointestinal hemorrhage	1 (0.6)	0
Glioblastoma	0	1 (0.6)
Hematuria	0	1 (0.6)
Hemophagocytic lymphohistiocytosis	0	1 (0.6)
Hepatitis B reactivation	2 (1.1)	0
Ischemic stroke	1 (0.6)	0
Lung disorder	0	1 (0.6)
Malaise	0	1 (0.6)
Metastases to bone	1 (0.6)	0

Myocardial infarction	1 (0.6)	0
Myositis	0	1 (0.6)
Nausea	0	1 (0.6)
Odynophagia	0	1 (0.6)
Pericardial effusion	1 (0.6)	0
Pneumonia bacterial	0	1 (0.6)
Progressive multifocal leukoencephalopathy	1 (0.6)	0
Pulmonary fibrosis	0	1 (0.6)
Pyrexia	1 (0.6)	0
Rash	1 (0.6)	0
Rectal adenocarcinoma	1 (0.6)	0
Road traffic accident	1 (0.6)	0
Sepsis	2 (1.1)	1 (0.6)
Squamous cell carcinoma	2 (1.1)	0
Thrombocytopenia	0	2 (1.1)
Thyroid cancer	1 (0.6)	0
Transient ischemic attack	1 (0.6)	0
Vomiting	0	1 (0.6)
Weight increased	1 (0.6)	0

Data are n (%) unless otherwise specified.
A, acalabrutinib; AE, adverse event; O, obinutuzumab; SD, standard deviation.

SUPPLEMENTAL FIGURES

Supplemental Figure 1. Investigator-Assessed Progression-Free Survival by Del(17)(p13.1)

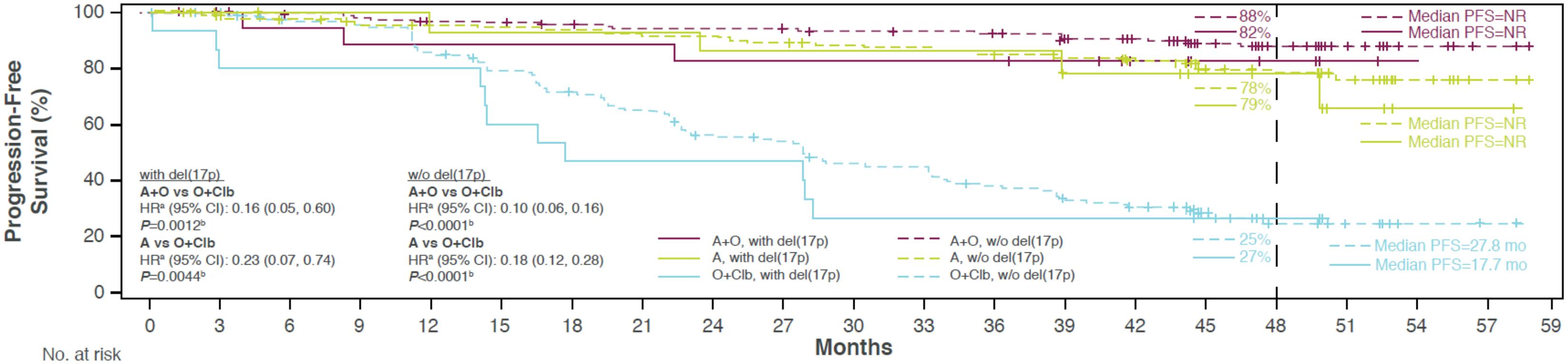

No. at risk																					
A+O, with del(17p)	17	17	16	15	15	15	15	15	14	14	14	14	14	13	10	5	4	1	0	0	0
A, with del(17p)	16	15	14	14	13	13	13	13	12	12	12	12	12	10	10	7	6	3	1	1	0
O+Clb, with del(17p)	16	12	12	12	12	9	7	7	7	7	4	4	4	4	4	3	2	0	0	0	0
A+O, w/o del(17p)	162	159	155	153	149	148	145	142	142	141	139	138	136	128	122	80	55	32	12	2	0
A, w/o del(17p)	163	152	149	144	143	142	140	137	137	134	130	129	124	120	113	72	55	32	15	3	0
O+Clb, w/o del(17p)	161	151	144	141	127	116	103	93	79	75	63	62	51	44	38	19	11	6	2	1	0

[a]Hazard ratio was based on unstratified Cox-Proportional-Hazards model. [b]*P*-value was based on unstratified log-rank test.
A, acalabrutinib; CI, confidence interval; Clb, chlorambucil; HR, hazard ratio; NR, not reached; O, obinutuzumab; PFS, progression-free survival; w/o, without.

Supplemental Figure 2. Overall Survival

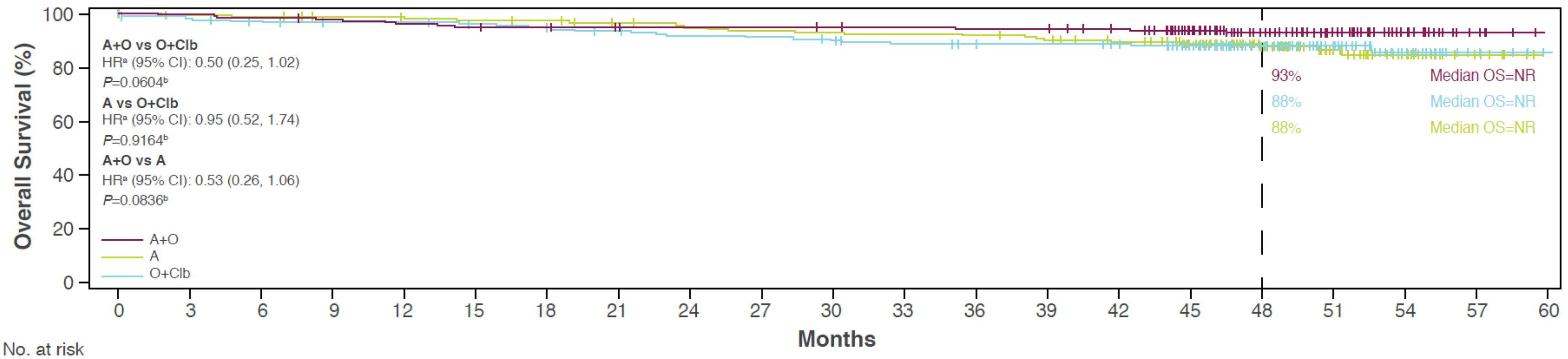

No. at risk																					
A+O	179	178	176	173	170	168	167	165	164	164	163	162	161	161	156	132	83	54	30	7	0
A	179	176	175	172	170	168	167	163	159	157	156	155	154	151	145	126	80	53	28	9	0
O+Clb	177	170	166	163	163	160	158	154	150	149	146	142	140	139	137	120	79	51	23	5	0

Note: In the O+Clb arm, 103 patients had disease progression, among whom 69 crossed over to the acalabrutinib arm and 34 did not.
[a]Hazard ratio was based on stratified Cox-Proportional-Hazards model, stratified by del(17)(p13.1) status. [b]*P*-value was based on stratified log-rank test.
A, acalabrutinib; CI, confidence interval; Clb, chlorambucil; HR, hazard ratio; NR, not reached; O, obinutuzumab; OS, overall survival.

Supplemental Figure 3. Investigator-assessed ORR (A) and MRD[a] status (B)

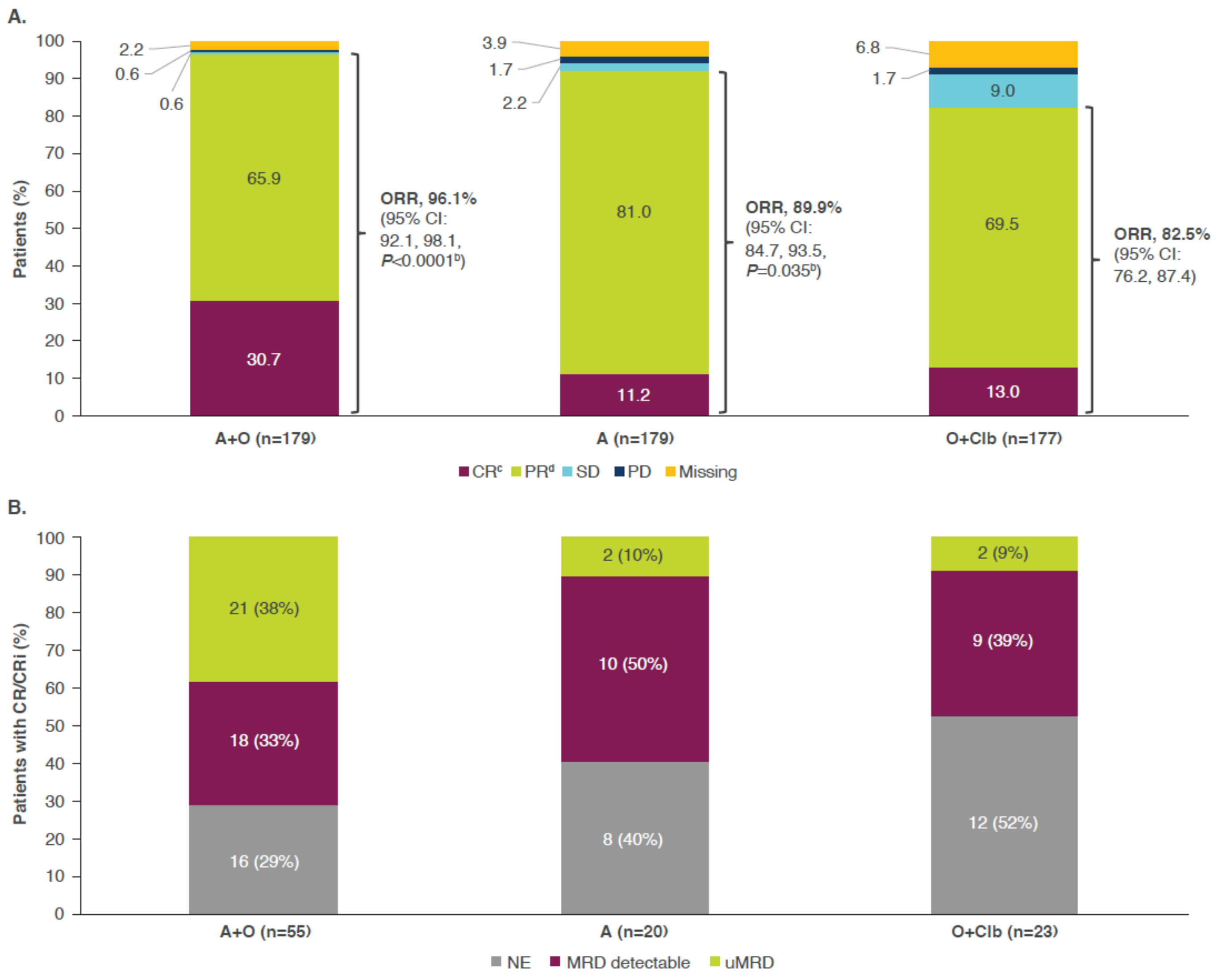

ORR is defined as achieving CR, CRi, nPR, or PR per the investigator or IRC assessment per iwCLL 2008 criteria at or before initiation of subsequent anticancer therapy. Peripheral blood testing to assess MRD occurred for patients with bone marrow-confirmed CR. Peripheral blood MRD status based on last two timepoints (most recent MRD assessments available by the data cutoff) in patients with CR/CRi.

[a]MRD was defined as the proportion of patients with <1 CLL cell in 10,000 leukocytes ($<10^4$).

[b]Based on Cochran-Mantel-Haenszel test with adjustment for 17p deletion status (yes vs no) vs O+Clb.

[c]Includes CR and CRi.

[d]Includes PR, nPR, and PRL.

A, acalabrutinib; CI, confidence interval; Clb, chlorambucil; CR, complete response; Cri, complete response with incomplete hematologic recovery; iwCLL, International Workshop on Chronic Lymphocytic Leukemia; MRD, minimal residual disease; NE, not evaluable; nPR, nodular partial remission; O, obinutuzumab; ORR, overall response rate; PD, progressive disease; PR, partial remission; SD, stable disease; uMRD, undetectable minimal residual disease.

Supplemental Figure 4. Cumulative Incidence Over Time for Any-Grade Events of Atrial Fibrillation (A) and Hypertension (B)

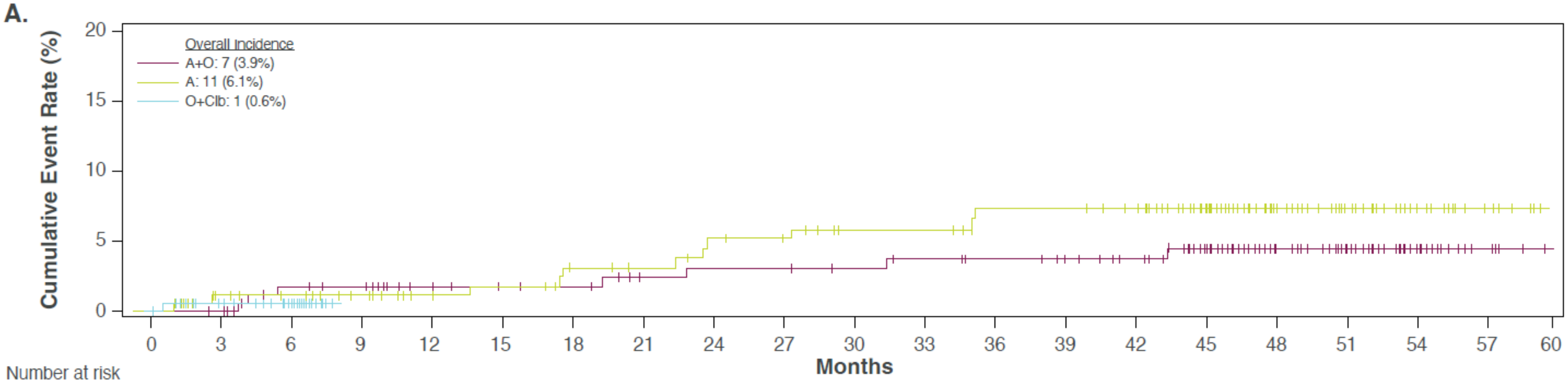

Number at risk	0	3	6	9	12	15	18	21	24	27	30	33	36	39	42	45	48	51	54	57	60
A+O	178	177	169	166	158	154	153	148	147	147	145	143	140	137	133	110	66	44	20	6	0
A	179	169	163	159	152	151	146	143	139	137	132	132	126	126	122	98	61	40	19	6	0
O+Clb	169	161	144	0																	

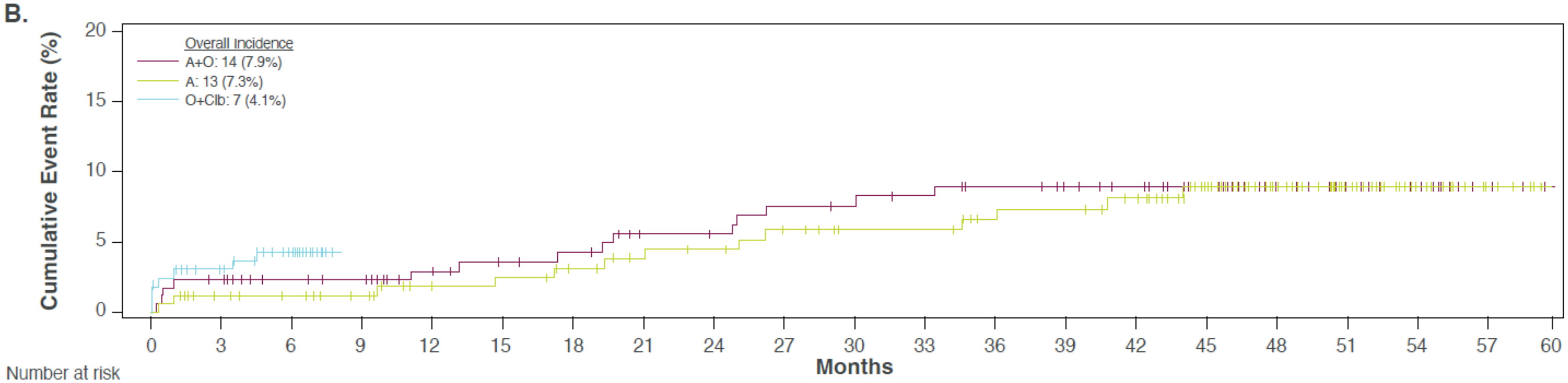

Number at risk	0	3	6	9	12	15	18	21	24	27	30	33	36	39	42	45	48	51	54	57	60
A+O	178	173	167	164	155	150	148	142	141	138	137	135	131	128	125	104	62	43	21	6	0
A	179	169	163	159	152	151	147	142	140	136	132	132	126	125	120	96	62	40	18	6	0
O+Clb	169	157	139	0																	

Treatment-emergent AEs during the randomization period only are included. O+Clb patients are censored at the last dose date of O+Clb +30, the start of new anticancer therapy, or the first dose date of crossover to A monotherapy -1, whichever came first.
A, acalabrutinib; AE, adverse event; Clb, chlorambucil; O, obinutuzumab.
